# Urban Digital Twins and metaverses towards city multiplicities: uniting or dividing urban experiences?

**DOI:** 10.1007/s10676-024-09812-3

**Published:** 2024-11-23

**Authors:** Javier Argota Sánchez-Vaquerizo

**Affiliations:** Computational Social Science, D-GESS. ETH Zürich, Stampfenbachstrasse 48, 8092 Zurich, Switzerland

**Keywords:** Urban Digital Twins, Metaverse, Participation, Social divisiveness, Human-centric planning, Collective intelligence

## Abstract

Urban Digital Twins (UDTs) have become the new buzzword for researchers, planners, policymakers, and industry experts when it comes to designing, planning, and managing sustainable and efficient cities. It encapsulates the last iteration of the technocratic and ultra-efficient, post-modernist vision of smart cities. However, while more applications branded as UDTs appear around the world, its conceptualization remains ambiguous. Beyond being technically prescriptive about what UDTs are, this article focuses on their aspects of interaction and operationalization in connection to people in cities, and how enhanced by metaverse ideas they can deepen societal divides by offering divergent urban experiences based on different stakeholder preferences. Therefore, firstly this article repositions the term UDTs by comparing existing concrete and located applications that have a focus on interaction and participation, including some that may be closer to the concept of UDT than is commonly assumed. Based on the components found separately in the different studied cases, it is possible to hypothesize about possible future, more advanced realizations of UDTs. This enables us to contrast their positive and negative societal impacts. While the development of new immersive interactive digital worlds can improve planning using collective knowledge for more inclusive and diverse cities, they pose significant risks not only the common ones regarding privacy, transparency, or fairness, but also social fragmentation based on urban digital multiplicities. The potential benefits and challenges of integrating this multiplicity of UDTs into participatory urban governance emphasize the need for human-centric approaches to promote socio-technical frameworks able to mitigate risks as social division.

## Introduction

The digitalization of every single component of cities has become a commodity in recent times, as the last iteration of the broader concept of smart cities (Cureton & Dunn, 2020). Urban Digital Twins (UDTs) aims to bring urban analytics to the next level. They represent more tangible, actionable, and concrete realizations of smart cities, which involve real-time data collection, analysis, modeling, simulation, prediction, and feedback into the city. Consequently, they are expected to help and enhance urban planning and operations to increase the quality and sustainability of cities. Overall, they involve data acquisition, analysis, visualization, prediction, and interaction expanded over the whole cycle of life of cities. Beyond technical and operational challenges, this article aims at repositioning the concept of UDTs by highlighting societal and experiential implications resulting from the rise of multiple co-existing examples that match metaverse visions that ultimately could reinforce difference realities and social divides.

### Many urban digital doubles: from models to twins

Digital Twins were originally coined within the context of manufacturing management (Grieves, 2014) and spatial exploration (Glaessgen & Stargel, 2012), echoing earlier visions of digital worlds (Gelernter, 1991). This initial definition already summarized the most important ideas of digital twins: mirroring physical assets in a virtual environment as accurately as possible to improve operations efficiency through real-time monitoring or prediction of the life cycle.

As such, they referred initially to closed, deterministic systems and environments where control theory postulates (Cardoso Llach, 2012; Wiener, 1961) could be easily applied as elements, interactions and limits of the system were well-defined. Hence, uncertainty and unpredictability issues due to complexity were manageable. However, over the years, the idea of digital twins has evolved and become more sophisticated, and therefore more capabilities are expected from them.

Digital twins have become common across multiple fields such as health, drug development, aviation, manufacturing, climate, agriculture or infrastructure management (Bruynseels et al., 2018; Errandonea et al., 2020; Fuller et al., 2020; Guo & Lv, 2022; Kanaga Priya & Reethika, 2024). This illustrates how they span through multiple scales, from molecules to people or the entire planet. Particularly, their application to the built environment has found a very fertile ground for development. It continues the tradition of urban modeling since the mid-twentieth century (Batty, 2009; Forrester, 1969; Hunt et al., 2005; Iacono et al., 2008; Lowry, 1964; Moeckel, 2018). It expands the last decade's industry and governance discourse around smart cities for increasing efficiency, optimizing processes, and improving the sustainability of cities using technology, sensing, and data. In this sense, digital twins can be considered their last iteration or even implementation (Deren et al., 2021).

Cities are particularly attractive for the development of digital twins. They are multi-scalar complex systems, the result of bottom-up and top-down processes, with a rich grand volume of data generated by intensive monitoring. This high availability of data is also the result of the convergence of well-established preexisting technologies to virtualize the physical built environment such as Geographical Information Systems (GIS), Building and City Information Modelling (BIM/CIM) technologies, ubiquitous sensing using the Internet of Things (IoT), integrated into 3D city models (Biljecki et al., 2015; Lehtola et al., 2022), and more recently everything merging into new standards for representing city elements, such as CityGML 3.0 (Kutzner et al., 2020).

However, different than in easier-to-control manufacturing environments, UDTs face bigger challenges. Cities are complex open systems, in constant change, which are usually defined as “wicked” problems (Rittel & Webber, 1973). Additionally, they involve not only tangible measurable and physical aspects frequently associated with the “static” built environment, but even most of what shapes cities is a myriad of intrinsically human socio-economic and cultural processes (Batty, 2024). Thereby, the definition of UDTs and their expected capabilities are still highly ambiguous (Depretre et al., 2022; Shahat et al., 2021).

These digital representations of cities and their built environment, physical counterparts are aimed to be linked through real-time bidirectional interactions and data flows, which make possible automatic data collection from the physical world, and feedback, or even change, from the digital representation back into the physical realm (Sepasgozar, 2021). This automatic feedback between physical and virtual realms is the main feature that distinguishes digital twins from other digital doubles such as GIS, BIM, and CIM (Masoumi et al., 2023; Shahat et al., 2021), or city control systems. The market-driven popularization of the UDT concept has rebranded (Kim et al., 2021) many of these originally considered digital shadows technologies (Sepasgozar, 2021), whose focus is rather in data aggregation, visualization, and communication. Now, they are seen as early-stage implementations of UDTs within larger recently proposed frameworks to try to systemize the whole ecosystem of digital doubles for cities (Gerber et al., 2019; Haraguchi et al., 2024; Kim et al., 2021; Masoumi et al., 2023; Raes et al., 2022; White et al., 2021).

**Fig. 1:**
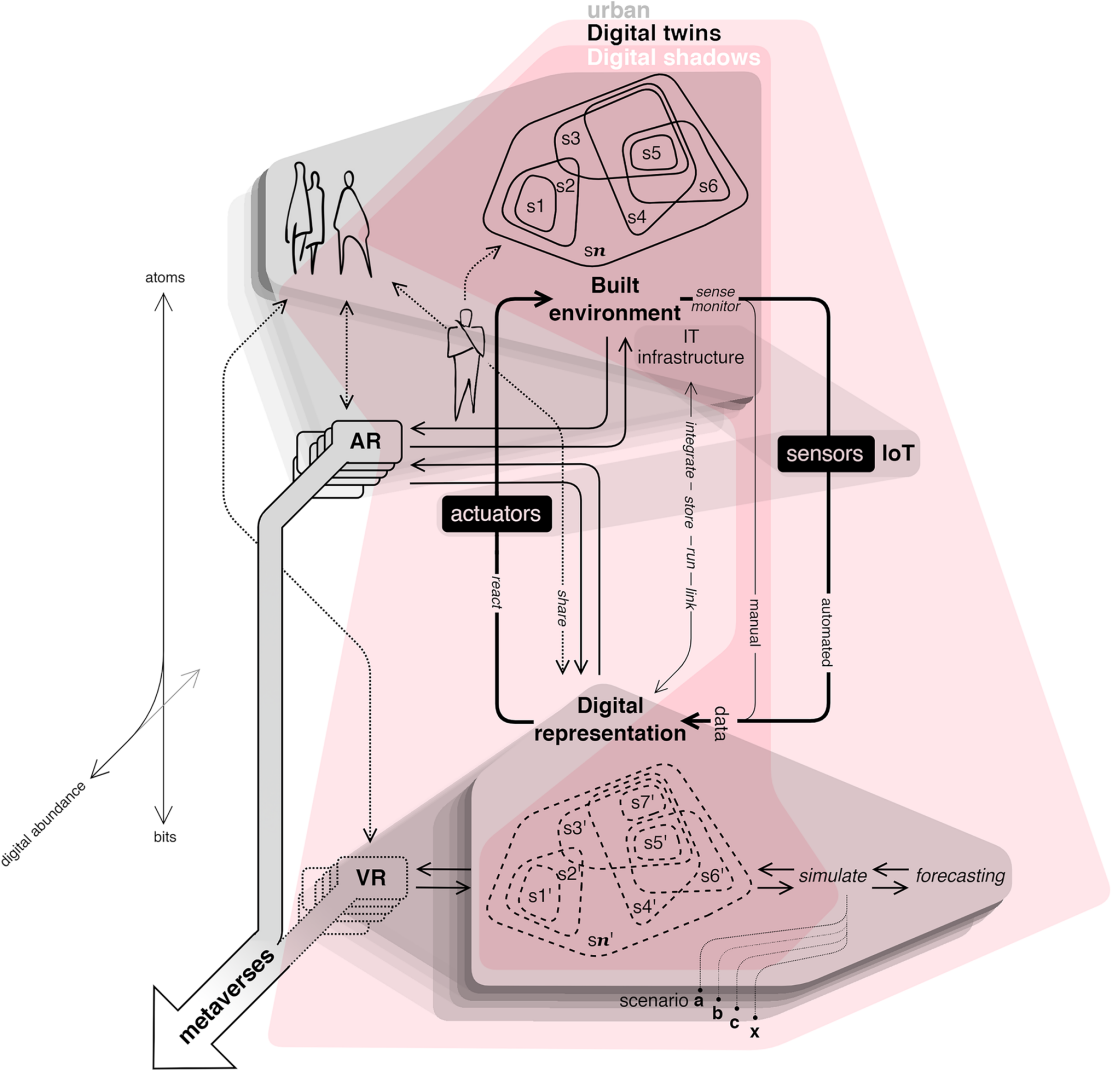
Digital doubles represent and expand the physical built environments made out of atoms into the digital realm of bits. The built environment contains a number *n* of intertwined by complex relations subsystems which can be digitally represented in *n’* models (Christopher, 1965). Digital shadows are the simplest digital representations, with limited capabilities and intractability. Digital twins are more accomplished, and sophisticated, with bidirectional near real-time and even potentially semi- automated data flows with the built environment. They expand their capabilities through simulation and forecasting of alternative scenarios different from the just mere representation of what is already. As part of the digital world of bits they participate and enhance the digital abundance: digital twins expand the already existing multiple visions and experiences of the built environment through expanded reality (XR) (e.g. augmented reality –AR- or virtual reality -VR-) creating simultaneous and coexisting layers of the same city in a collection of urban metaverses. Figure based on (Saracco, 2019)

Consequently, the term “digital twin” has become ambiguous, and it spans from more or less advanced expanded implementations of city models to the most recent approaches, which incorporate real-time information feedback loops and complex simulations of socio-technical systems. We can consider all of them as urban digital doubles, being the former models and digital shadows, and the later, gradually closer to the idea of proper digital twins (Fig. 1 and Table 1). This most accomplished vision of digital twins requires the integration of simulation and optimization with the processing of massive volumes of low-latency data, which involves ubiquitous sensing and great computational power (Liu et al., 2021; Wang et al., 2019). However, no example exists yet that integrates all the expected features, although academia, industry, and government may envision to grand scale digital twin as the ultimate goal (Lim et al., 2022; Nativi et al., 2021). The rest of the article will focus on these more advanced, interactive, complex, and still-to-be-developed UDTs.

**Table 1:** Summary of main differences between traditional urban models (e.g. digital shadows) and the proposed framing for UDTs

Dimension	Digital shadows (traditional urban models)	Urban Digital Twins (as presented in this article)
Data flow	One-way data flows from the physical world to the digital representation	Two-ways data flow with (near) real-time updates and feedback loops between the physical realm and digital representation
Purpose	Focus on visualization and accurate, static replication for observation	Dynamic models for simulation, scenario planning, and active management
Representation	Singular, isolated, and centralized representation of urban systems	Dynamic, multi-dimensional models integrating various systems, data, and scales
Stakeholder involvement	Limited involvement, usually technocratic and corporative oriented	Participation of multiple stakeholders, including policymakers and citizens
Impact on Urban experience	Minimal focus on user or social interaction	Actively able to shape differential and tailored urban experiences, promoting either customization, inclusion, and accessibility or social divisiveness
Societal impact	Minimal focus on societal consequences, limited to the scope of the policies that they may inform	Politically and socially loaded, with the potential to create fragmented urban realities

### Coupling with reality and the emergence of metaverses

There could be some fuzzy agreement about the general capabilities that an UDT should tend to have, differentiating them from other digital doubles of cities: “integrated models based on 4D (i.e. spatio-temporal), relational and sensor interoperable, open data which could be used for simulation and prediction at different spatial and temporal scales” (Ketzler et al., 2020). Nevertheless, there is still a very wide range of possible levels of accomplishment for a digital twin (Evans et al., 2019). At the same time, it is more unclear the actual nature and expectations of a digital twin regarding coupling, interaction, feedback, and closeness to the physical reality. On one hand, in the short-term and high-frequency scope, (Batty, 2018) a close coupling is expected between the physical reality and its representation in such a way that the collection of data should inform and be processed automatically, and in real-time by the model to provide immediate feedback that can support constant readjusting and optimization of the physical reality. This approach would be more related to continuous operations and management and requires a tight coupling between physical assets and their representation (Tomko & Winter, 2019). As a result, the digital twin can be considered as an integral part of the physical system, now rather a cyber-physical-social system. A complementary perspective is the speculative and longer-term, low-frequency city, in which the virtual representation needs to detach from reality to explore alternative, never-existing, scenarios which ultimately can inform future operations. As such, a tight coupling, a mere mirrored representation of the physical reality would limit the exploration of alternative scenarios, which precisely requires detaching from the physical reference (Batty, 2018). Hence, this understanding of UDTs represents a first methodological divergence or split from reality.

This multiplicity of urban scenarios can be particularly enhanced by Extended reality (XR), whether in the form of virtual (VR), mixed (MR), or augmented reality (AR) (Rosenberg, 2022). Expanding the cyberpunk original science fiction concept (Gibson, 1984; Stephenson, 1994), urban metaverses can be understood as several collective virtual shared spaces mirroring and expanding existing cities where people can interact, work, play, and socialize in real-time using avatars and digital identities with a plausible sense of presence (Ball, 2022). They can take the form of different XRs, whether as completely virtual environments or as virtual layers on top of the physical reality that is augmented, while they could consist of multiple coexisting and interconnected XRs (Rosenberg, 2022) (thereby, we would talk rather of a metaverse of metaverses). Every stakeholder involved in city planning can come to the urban metaverse, participate, and exchange ideas to reach a consensus (Dembski et al., 2020; Helbing et al., 2023). These spaces are characterized by their persistence, interoperability, and the ability for users to create and influence the environment and content within them in an open-ended way. Thereby, urban metaverses are all these possible expansions of the built environment made possible by the interplay, aggregation, and federation of UDTs and XR technologies to create a range of variations over the built environment in several dimensions as urban digital multiplicities:

- From augmented ones to fully virtual ones,
- From fully individually tailored to increasingly co-shared with other people,
- From tightly coupled with reality to sandboxes able to anticipate and test new speculative settings, policies, and designs for cities to be potentially generalized.

### The limits of coupling, representation, and predictability

Any of these digital representations will be bounded by the very own nature of the data used (Helbing & Argota Sánchez-Vaquerizo, 2023) and by the decisions and methods involved in the curation of this data. A mere accumulation of big data processed by opaque and hard-to-understand and interpret machine-learning algorithms falls short. The lack of consideration of complex effects, interactions, and interdependencies (Caldarelli et al., 2023; Grieves & Vickers, 2016) contributes to the limitation of the digital representations of cities. Cities are the result of the interplay of bottom-up and top-down processes. As such, a system aiming at representing, analyzing, and forecasting the future of urban environments would try to get as close to the physical environment as possible. While assuming the simplification of our models (Batty, 2021) our current data-driven approaches over-represents physical assets and rely heavily on data analytics tools, perpetuating a restricted vision of cities as if they were soulless, asocial machines.

Considering simultaneously different models that are at least partially valid or useful may help to overcome the limitations of representing and modeling socio-economic, human systems of the physical world. (Helbing, 2013). Also, this incorporates multiple worldviews and approaches to problems which can be hard to be fully formalized and hence intrinsically wicked (Batty, 2021; Rittel & Webber, 1973). This pluralistic modeling could consist of many parallel, federated digital twins able to provide a more accurate picture of the systems, their interactions, and their future, by encapsulating the diversity, conflicts, and tensions of our real world (Gerber et al., 2019; Hudson-Smith et al., 2022; Page, 2018). While the understanding of human socioeconomic processes has improved over the last decades, it is still needed to define and model the relations between physical and socio-economic representations (Batty, 2018). Particularly, the inclusion of human, social, cultural, and psychological factors, hardly quantifiable and still fundamental for the quality of human settlements, is still a challenge in the representation of urban systems. However, it may be considered impossible to predict and forecast holistic future scenarios (Cureton & Dunn, 2020).

The representation and conceptualization of the city would benefit from:

- Combining data-based and hypothesis-based approaches based on theory linked to complexity science (Caldarelli et al., 2023), and
- Updating and enriching the way they codify and represent components, processes, knowledge, and relations happening in cities. Semantic representations (Chadzynski et al., 2022; von Richthofen et al., 2022) in digital twins developments can enable a better, richer, and more aligned representation of reality with human understanding.

Semantic approaches to data representation can unlock new possibilities for raw data used currently in urban planning, modeling, and urban codes:

- It enables making raw data more understandable, and ultimately, operationalizable, and
- It allows expanding the possibilities of their exploitation to address domain expert questions and challenges.

Overall, it faces some of the current issues on how much data is needed to accurately grasp, represent, or depict urban life. The question may be on how we represent and encode that data. This high-level approach aims to generate agnostic graph knowledge platforms with a potential global scope (Akroyd et al., 2021).

### The role of people: participation

The role of humans in the development of UDTs, whether as operators, people-in-the-loop, or even simply as end-users, collaborators, or sources of information is one of the main challenges (Abdeen & Sepasgozar, 2022). People may have different considerations than planners and designers regarding the built environment, which adds to the unpredictability of environments that have not been planned for the unplannable (Gram-Hansen, 2017). In this sense, there is a public interest in engaging citizens in the utilization of UDTs to enrich, analyze, and forecast cities' evolution (Yamu et al., 2023). It is promoted by international good practices and it is an increasingly common trend in cities as shown in many participatory and co-creation processes as fundamental for sustainable urban development (Bouzguenda et al., 2019). If cities are designed for the well-being of the people they house, citizens should be able to participate in their planning. Indeed, digital twins can be potentially examined and experienced by the general public to provide feedback on the proposed changes in the city (White et al., 2021). However, most of the development of digital twins has focused on the physical aspects of cities, with little role of people and social, economic, cultural, and behavioral processes (Batty, 2024). Also, we cannot disregard the well-known challenges of citizens' participation in planning processes in cities even out of the context of digitalization, which usually includes misrepresentation, instrumentalization, and other forms of flawed participation (Blundell Jones et al., 2013). Alternatively, including citizens’ participation in UDTs can be used to increase situation awareness about future plans while providing an understandable interactive way that can overcome engagement obstacles for consultation processes (Ketzler et al., 2020).

People can play different roles in this process and may appear in different stages. From an interaction point of view, they can be the end users of a given framework, whether just as receivers of higher-quality information or actively participating in planning and decision-making processes. In the former, people can be simple users of digital frameworks and platforms for useful information; they can interact and elaborate on information together with other citizens. In the latter, they can be even the curators and creators of crowd-sourced data for these systems, if not really engaging and being part of planning support (Batty et al., 2012).

As any element is subject to be conceptualized, coded, analyzed, and planned, people are not only the prosumers and evaluators of data, frameworks, and resulting policies and planning decisions. They are also active constituents of the built environment whose behavior and cognition are subject to be analyzed and modeled. Their interactions with the built environment are usually not sufficiently represented and understood in our existing big data, from a cognitive, psychological, and cultural point of view. As active agents of urban systems, people interact in different ways between them, with other elements, with the environment, and with the tools available. XR has been proven to be very handful for research behavior in the built environment and to enhance the role of citizens as active agents of innovation, inclusion, and social development (Dembski et al., 2020; Hudson-Smith & Batty, 2023; Hudson-Smith et al., 2022; Sanchez-Sepulveda et al., 2019; VU.CITY, 2021; White et al., 2021). Also, mirroring the diversity involved in city shaping, and building successful and actionable UDTs demands the collaboration of multiple stakeholders with diverse expertise (Allam et al., 2022) and reflecting local knowledge (Nochta et al., 2021). It means, co-create tools for co-creation.

The use of computer intelligence to expand the capabilities of people to participate in the planning of changes in cities goes beyond mere interaction. One of the challenges of participation in urbanism, as in any deliberation and decision-making process, is the sharing and combination of different agendas, preferences, and opinions. Diversity is a feature of cities. Divergence is embedded in the experience of the city, not only from opinions and preferences. The same urban context, the same built environment, the same location, and features will be evaluated, appreciated, and perceived differently by different individuals (Pocock & Hudson, 1978). The current development of Artificial Intelligence (AI) aims at supporting democratic participatory processes from where people could expose and receive feedback from their fellows mediated by computational intelligence. It means digitally assisted deliberation and debate, to align opinions and preferences (Zaremba et al., 2023). From the understanding that (urban) digital twins require bidirectional flows of information, the role of stakeholders and people, in general, is even more important. Within this perspective, the feedback and directionality can be understood from a very computational, data-driven perspective. It is, in tangible and computable data flows between the physical reality and the virtual representation. However, it can be seen from a more cognitive, policy, and social perspective: directionality can be also informing people and interactions between human “operators” involved in the decision-making process and the virtual mirrored representation. In this context, the actuators would be the humans being affected by the digital twin.

## Examples of Urban Digital Twins

The generalization of the use of UDTs in the last years has boosted the number of examples illustrated in science and industry. Despite the confusion of the term and the very early stage of their development, they can be conceptualized and defined based on their application data, inputs, data processing, visualization, outputs, and actuators in the physical environment (Ferré-Bigorra et al., 2022).

The selection of urban digital doubles (Table 2 in the Appendix) tries to expand the scope of analysis on UDTs beyond normative and technical definitions and exhaustive scholarly reviews. The aim of this state-of-the-art review (Grant & Booth, 2009) is to analyze existing tools, implementations, and frameworks while expanding the research scope by adding examples that can be considered unexpectedly as equally or even more accomplished realizations of UDTs. Therefore, it bridges the gap between scholarly production and grey literature. Above all, it tries to add a new perspective on UDTs to highlight how they can be related to human-centric planning. For this purpose, the analysis does not focus only on scientific literature and does not seek for completion of exhaustiveness, as of a systematic review, of all the digital twins recorded in academic texts such as other recent reviews on the subject (Alva et al., 2022; Boje et al., 2022; Ferré-Bigorra et al., 2022; Jeddoub et al., 2023; Ketzler et al., 2020). Therefore, it includes well-known published academic examples, together with other industry and government-related solutions to illustrate trends and directions of technology and practice with a focus in these examples that involve citizens and other urban stakeholders’ participation.

The selection of examples is driven by a practical and human-interaction perspective. Therefore, it is limited to functional, interactive, concrete, and situated implementations of UDTs, as gathered from recent reviews. Functional refers to examples that go beyond theoretical or abstract framework proposals, have a clear case study, and a purpose. Interactive refers to allowing for some type of people’s interaction, e.g. citizens, non-experts. Therefore, prototypes or frameworks which have not been publicly available, or whose use has been restricted to only in-house experts, officers, and governments are not included. Also, it includes implementations of XR examples that are coupled with the physical environment (i.e. reality-based) and which engages with citizen participation. Concrete and situated refers to examples that are linked and paired to a physical, defined, and located environment. Hence, for instance, metaverse applications developed in VR without a connection to a location are not included.

From the selected examples, some common trends can be identified (categorized and summarized in Table 3 in the Appendix). Firstly, we can identify earlier implementations of open UDTs developed by city governments such as those of Amsterdam, Antwerp, Boston, Helsinki, Rotterdam, Victoria, Vienna, Zurich, and Zwolle, which are rather expansions on previously available GIS data visualization tools (Boston Planning & Development Agency, 2018; City of Amsterdam, 2020; City of Helsinki, 2019; City of Vienna, 2021; IMEC, 2018a, b; Lehner & Dorffner, 2020; NSW Government, 2022; Rotterdam, 2021; Ruohomaki et al., 2018; Schrotter & Hürzeler, 2020; Victoria State Government, 2022; Yamu et al., 2023). They are an expansion of policy and governance concerns. They add at least 3D models (Biljecki et al., 2015), including building, vegetation, and topography, which enable in some cases visibility and sunlight studies. Their availability on open websites makes already existing open data sets more accessible and understandable for people by creating integrated and interactive visualization tools. Therefore, they represent also extensions of already undergoing open data initiatives hosted by many of these cities. The interaction is pretty much limited to making accessible and transparent planning and some environmental information to people. Citizens have easier access to useful information for their daily concerns or just for already existing public consultancy periods for urban planning. Hence, the feedback is just based on information received by citizens that may enact further action or change their opinion to activate other counterbalances in the existing political and bureaucratic framework. These cases are slightly closer to the traditional urban models and digital shadows (Fig. 1 and Table 1).

The examples from Antwerp, Gothenburg, DUET in Athens, Plsen and Flanders, Madrid, Rennes, Rotterdam, Singapore and some Australian states integrate dynamic data into the city model (Ayuntamiento de Madrid, 2024; Government of Singapore, 2018; IMEC, 2018a, b; NSW Government, 2022; Queensland State Government & Terria, 2021; Raes et al., 2022; Rotterdam, 2021; Victoria State Government, 2022; Visual Arena, 2022). This data could come from people's behavior, goods movement, and energy flows in the city such as the ones associated with mobility or logistic networks; or from IoT urban sensing infrastructure, such as in the case of environment monitoring. Their scope falls between data aggregation and visualization, and advanced planning and urban management for improving city operations (e.g. mobility, urban design, policymaking, or resource allocation) with different levels of stakeholder engagement, toward the development of more complex platforms for coordinating and aggregating different applications, models, and twins (Ayuntamiento de Madrid, 2024; Government of Singapore, 2018) even at larger geographic areas such as SMART Zwolle (Yamu et al., 2023), or even entire countries like Japan (MLIT, 2023). Precisely, smart energy is one of the domains that attract more attention for the development of UDTs, probably due to their similarities to controllable manufacturing environments. This is the case of the platform developed for Cardiff (Boje et al., 2022; Cardiff University, 2024), and pilots for smart districts and smaller sections of a city (Alva et al., 2022) like in the Georgia Tech campus (Xu et al., 2019) or new developments such as Nottingham’s Trent Basin (IES, 2021; Strielkowski et al., 2022), and Aspern in Vienna (ASCR, 2015). Despite their focus on energy optimization, they highlight the importance of user interaction. Other applications, such as in the case of Newcastle, focus on emergency response (Wolf et al., 2022). The examples of Pisa and Turin show the two-fold need to gather data of interest for people and engage them in the use of these tools was also a common enhancer of people's participation in creating and interacting with these solutions (Bacco et al., 2017; De Filippi et al., 2020). This allows for a more sophisticated exploration of complex aspects of cities, increasing the legibility of not-so-obvious aspects of urban dynamics (Mohammadi et al., 2020).

While keeping the focus on governance and policy-related aspects, other urban digital doubles have a stronger focus on people participation. While the UDTs of Dublin Docklands, Herrenberg, Gothenburg, Kalatasama, Nancuiping Park, Zwolle, and the Pasymo project for a few cities in Brandenburg are built on top of existing geospatial data expanded and visualized in a 3D city model, they aim at people’s interaction to provide feedback from citizens’ local knowledge (König et al., 2017) and their preferences (Airaksinen et al., 2019; Dembski et al., 2020; Luo et al., 2022; Priebe et al., 2019; Visual Arena, 2022; White et al., 2021; Yamu et al., 2023). These examples are not only representations of existing geospatial data but elaborations of dynamic data layers, such as simulations resulting from mobility flows or urban sensing. Also, they allow for the representation of alternative plans and non-existing scenarios more effectively for people and stakeholders to express their different opinions and concerns. Hence, they need to create additional ways of interaction to elicit people’s responses. For this purpose, some projects for Barcelona, London, and the digital twins for Herrenberg, and Dublin Docklands implement human–computer interaction methods using immersive environments in XR that facilitate engaging visualizations of abstract aspects of urban life (Dembski et al., 2020; Hudson-Smith & Batty, 2023; Hudson-Smith et al., 2022; Sanchez-Sepulveda et al., 2019; VU.CITY, 2021; White et al., 2021). The design of interaction is a key aspect of these examples, as it ensures the coherence of the linkage and the robustness of the learning and co-creation process (Liu et al., 2022).

A different approach is followed by other immersive developments for participation that proposed urban metaverses that focus on marketing, events, tourism, and local business through interactive gamification like in the case of Santa Monica Metaverse (Allam et al., 2022). Seoul's metaverse project (de Almeida, 2023; Seoul Metropolitan Government, 2023) and AR Incheon (Um et al., 2022) have leveraged digital twins to engage people in novel ways with the city, expanding services and enhancing experiences. Although still in the early development stages, their primary aim is not urban planning. Also, their coupling and feedback loop to physical reality is not designed, although it may be expected to happen just by motivating new visitors and supporting marketing and tourism-related activities like in the case of Benidorm (Six3D & Ayuntamiento de Benidorm, 2023) or tokenized economy aiming at having positive business outcomes such as in the case of Dubai (Metaverse Dubai, 2022).

One of the most common ways to enhance participation and engagement in urban issues is serious games (Poplin, 2012), an approach that has been tested in Dadaocheng, Helsinki, and Gothenborg (City of Helsinki, 2019; Ruohomaki et al., 2018; Visual Arena, 2022; S. Wang & Vu, 2023). The Royal Ports of Stockholm, Hämeenlinna, Olomouc, and Svit have gone a step further by implementing gamified strategies using directly existing video games to create UDTs, simulate the dynamics of the city, and use their already built-in capabilities and interactions to engage people in the planning process and integrate collaboratively their preferences in the design (Pinos et al., 2020). While these digital twins do not deploy a full ecosystem of consistent, massive, and immersive metaverses, they are operationalizable and successful examples of the use of participation in digital twins for planning.

The use of existing tools such as games across different locations like in the case of “Cities: Skylines” (Pinos et al., 2020), or even Minecraft (Wang & Vu, 2023), highlights the importance of interoperability of these frameworks. While acknowledging the importance of digital twins, the lack of standards in the specifications of data, encoding of information, architecture, format, and interactions, among others, is one of the main limitations for the generalization and scalability of these technologies (Lei et al., 2023). Most of the presented examples are based on the exploitation of isolated data silos and are ad hoc solutions for a particular set of locally situated problems. This endeavor drives the development of general frameworks to create digital twins covering from data acquisition to human-centric modeling and end-user interaction which can be replicable across multiple locations (Raes et al., 2022). Developing these standard frameworks for UDTs also requires conceptualizing their design from a socio-technical perspective and understanding the current processes of planning and policymaking at a local level as illustrated in an updated version of the UDT from Cambridge from a social-technical perspective (Nochta et al., 2021).

A good example of the idea of global scalability and coverage from a common framework and clear standards and interaction rules aiming at a global audience is Google Maps (and in fact, all the suite of geospatial-related Google products such as Google Street View, Google Earth, or Sunroof). Although it is not frequently considered, when Google Maps is analyzed from the perspective of an UDT, it covers many of the expected features (West, 2021). It gathers and integrates tons of geospatial data, as well as in real-time (e.g. traffic, business activity, and people concentration from Android user habits and behavior). The interaction rules are defined, and people get immediate feedback about the current state of the physical environment while accessing useful information for daily life. It operates as a platform supporting additional applications, which enhance planning, visualization, immersion, or design capabilities. Additionally, end-users can provide feedback, opinions, and additional data, although it is mediated, moderated, and filtered by the service owner. Also, it allows for forecasting future scenarios based on past learned data. At the very end, people can plan and change their behavior (within the scope of the available data and interactions, i.e. changing a route, the departure time, or visiting a shop) accordingly. Consequently, even within the relatively limited scope of the software, it is probably a glimpse of how a global digital twin may look in the future.

However, there are more obvious, invasive, and disrupting initiatives of intensively data-driven cities that aim to monitor the whole life cycle of the built environment under these human-centric data-driven systems for sensing, modeling, mapping, and accounting such as the failed attempts of Alphabet to redevelop Toronto’s Quayside (Sidewalk Labs, 2017). Under the premise of people using their own data to improve the functioning of the city, they proposed a pervasive monitoring and data exchange to automate planning, services, and governance. The core of the proposal was the development of planning and governance automated through data-driven outcome-based code, which embedded a whole replica of citizens moving and behaving in a simulation of the city. This raised concerns regarding accountability, transparency, and privatization of public governance processes. It proposed replacing the existing socio-political organization in the city with some sort of data-driven and market-signal system. Eventually, the initiative was abandoned because of concerns regarding privacy, governance, accountability, and lack of investment support (Artyushina, 2023), which highlights the inherent challenges to converge planning, governance, and market-driven interests within the context of UDTs as metaverses that effectively affect daily life.

## Discussion

### Future realizations: from twins to metaverses

Real UDTs with automatic informational loops between physical and virtual realms have not been accomplished yet. It is still a concept under development and lacks many essential components to urban and human life. The considered cases in the previous section only include partially one or a few of the expected features of UDTs. Although some promising examples start to exhibit the multiplicity of scenarios enhanced by participation, the human factor is still a challenge to be effectively integrated into digital twins in the endeavor of overcoming a purely engineering a technical perspective. While our massive big data promises to provide more accuracy and better predictions, we need to take into consideration that these assumptions will fall short: data is limited, bounded, partial, and mediated by definition, which limits ultimately the way we represent our physical world and couples with the embedded uncertainty of such complex systems (Caldarelli et al., 2023). Urban systems, and cities, are not deterministic machines (Mattern, 2017).

The urban metaverse can be understood simultaneously as the expansion and as a consequence of UDTs with the inclusion of diversity from people and societal interactions (Lv et al., 2022), i.e. interactions with all the digitized elements and between the users.

The construction of its functionality, trustworthiness, and purpose relies on three main areas:

(i) As a cooperative tool to support governance and planning, urban metaverses inherit features of UDTs as an evolution of the latter caused by social diversity. They are very accurate digital representations of the built environment, open to the participation of different stakeholders. They are distributed, interoperable, open, federated, decentralized, scalable, and synchronized ecosystems of urban applications able to facilitate the integration of multiple data sources and formats to improve services, operation, and planning of the city. This is possible by offering a holistic vision of the city by including the representation of urban assets and dynamics engaging with urban complexity, while facilitating interfacing with the multiple constraints and regulatory conditions that usually make it troublesome to navigate policy and planning processes, particularly for non-experts. This includes creating virtual scenarios suitable for multiple applications for analysis, simulation, and evaluation of infrastructures, including safety concerns. The integration of predictive models allows for comparing different policy and planning strategies such as urban sustainability. Finally, they are synchronized with the physical city in collaboration with human intelligence to respond in real-time to specific and personal needs, and ultimately actuating and causing changes in the physical reality.
(ii) As an expander and diversifier for reality, citizens and other stakeholders can not only envision possible futures but respond to them accordingly. As a result of the discussion, exchange, and construction of alternative futures, UDTs will lead to urban metaverses that could expand effectively the physical reality (XR). Thereby they could modify how people live and interact with the built environment, dividing the use, perception, and experience of the built environment either for exploration, testing purposes, or for providing an adapted, customized, or fitted environment to particular needs, preferences or even capabilities (Kuru, 2023).
(iii) Finally, it is possible to consider new and unbounded possibilities for economic and social activity. This approach is inherited from existing examples of decentralized virtual worlds for leisure and business, although their convergence with governance goals is still to be defined either as just boosters of a new economic activity sector or as part of a deeper process of tokenization of physical assets in a supposedly more decentralized financial system.

If we also consider the metaverse as a 3D extension of the Internet, we can anticipate some trends based on what we are experiencing on a more mature technology as the web. The Internet operates to some degree as a sandbox where alternative scenarios, lives, frameworks, and overall, ways of living, are tested in a virtual setting before they materialize in physical reality. Similarly, in the built environment where the same physical locations are experienced and perceived differently, the Internet makes even easier these effects. People perceive and interact differently with the same hypertext protocol, the same infrastructure, the same code, and the same information that supports the whole web, and not exclusively because of individual cognition differences that lead to different personal experiences. Banners and ads, product recommendations, or news and social media content and feeds are personalized. While all this raw information is available to everyone in the same infrastructure, people experience different partial subsets defined by code.

This anticipates how the interaction of citizens with the built environment could be with a more intensive and pervasive mediation of software and code, whether it is through modeling, digital twins, or immersive XR environments. Divisive and differential experiences and interactions in our inhabited environment (whether physical or virtual) will be more frequent. In fact, the same physical built environment supports different individual and collective experiences of the space. The same environment is perceived differently by different people. Moreover, the built environment is normatively segregated for being used by different groups of citizens at different moments.

In the coming years, the use of smartphone apps and headsets will facilitate to augment our physical environment, hence expanding its affordances (Gibson, 1979). On one hand, adapting and customizing in real-time and continuously the outlook of our built environment will become common. Almost like already existing instantaneous filters used by smartphone apps, it would be possible to adapt our space to our needs and preferences seamlessly (Lopez Rodriguez & Pantic, 2023). We could imagine a hyper-media layer where useful, personally curated information regarding your daily chores that only you can see is displayed overlying the physical reality. Existing devices that allow us to visualize privately our own media or workspace merged in the physical space that we share with others are already outlining this possibility. Also, current BIM, construction management, and industrial XR applications can guide, anticipate, and help to visualize hidden or future components and layers of information. Or cultural heritage applications that currently are helping to record artifacts and rebuild past environments and settings will converge with educational and tourism-oriented services to show lost physical environments. On the other hand, it makes it possible for unequal levels of augmentation of the environment that can provide advantages only to those who can access them. This vision of an augmented-reality metaverse is not new and has been already pictured years ago (Matsuda, 2016).

Whether in a purely virtual-based metaverse or for extending physical reality, it would be possible to create digital realistic replicas of any given environment or any element effortlessly. Beyond industrial mass production, it provides even further endless possibilities to generate versions and alternatives, which can adapt to personal needs or preferences. This post-mechanical digital reproduction deepens the shift in creative production regarding its sociocultural value, and its political role (Walter, 1936), now also revealing social insights hidden in data (Kalpokas, 2023). At the same time, this digital abundance allows expanding the built environment, and potentially even could replace physical construction, making some new buildings unnecessary, and thereby saving resources (Trickett, 2023). The development of increasingly more sophisticated, realistic, and embodied interactions and feedback methods would be critical for their success. Altogether, any type of metaverse would enhance the augmented diversity of a city with very differential final environment realizations over the same infrastructures, which here, literally, refer to the underlying physical reality that supports presence.

Furthermore, the challenge is not only to design a cyber-physical environment with meaningful interactions for people. Within the domain of human–computer interaction, there are still important challenges. The generative potential of AI is still in its early phases, as it is the way that humans can interface with machine intelligence in increasingly closer forms to natural language (Kumar et al., 2023). Leaving aside the interpretability of the opaque undergoing algorithms that can generate humanly plausible results and interactions, the next step is mixed media. Large Mixed Models (LMMs) (Yang et al., 2023) will be able to connect human cognition, expressiveness (and ultimately wishes) into computer-readable information that could be transformed in any media or humanly meaningful stimuli, whether we refer to image generation, sound, haptics, among others. The combination of the metaverse’s ability to generate endless copies and variations of a given scenario through augmentation and virtualization, along with a seamless connection of human cognition (including speech, or even thought) to generative AI opens a future of endless possibilities.

**Fig. 2:**
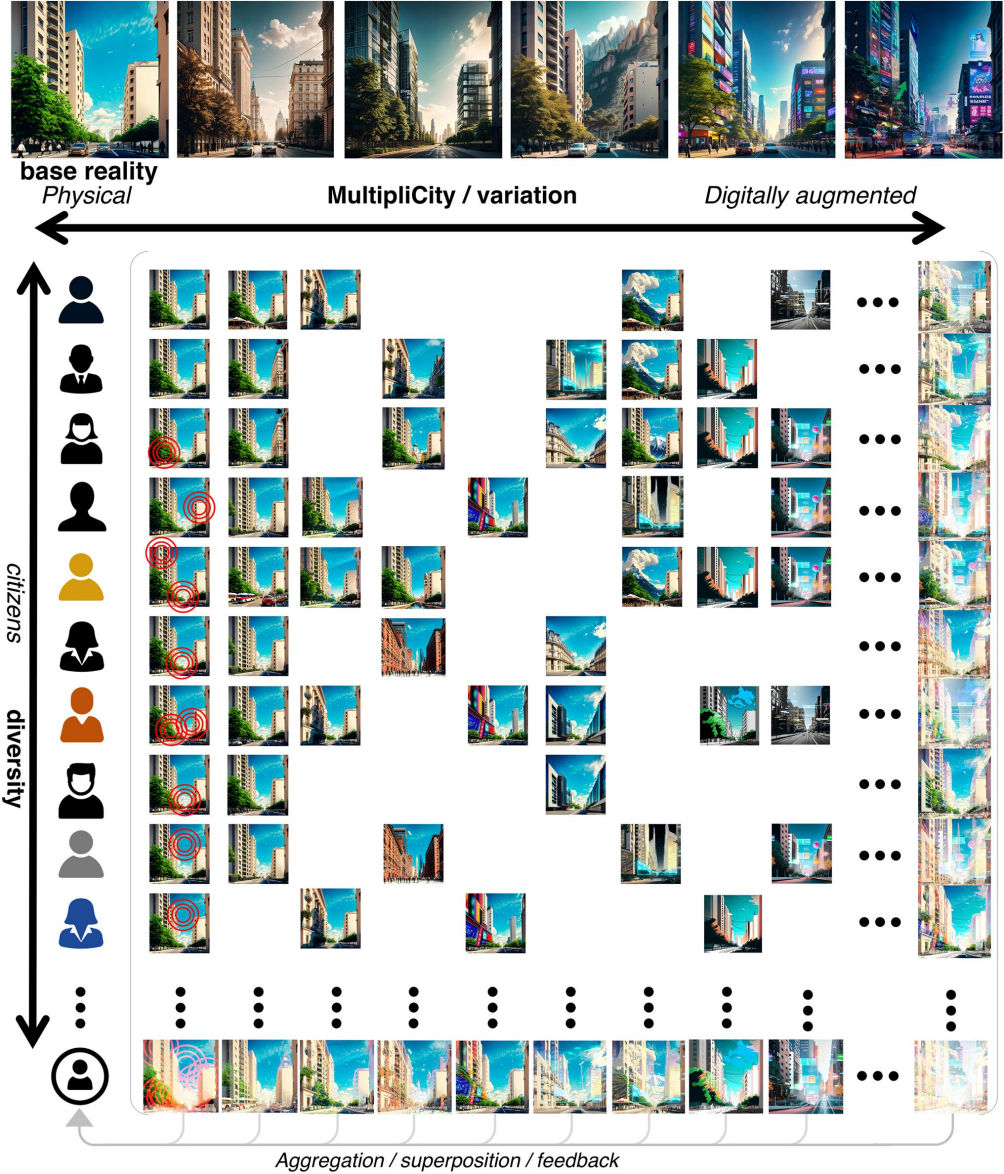
Multiplicity of cities: urban digital multiplicities. From the same physical environment, endless variations may be accomplished. They can be caused by cognitive and psychological differences between individuals (vertical axis) or by on-purpose variations (horizontal axis) that can help to build up collective intelligence in the configuration of future cities. Some of them will be unique, others will be shared. Simultaneously, they can enhance segregated experiences of the city breaking apart the common sharing of the urban environment. Variations of the same scenario have been generated using Adobe Firefly

Every single individual may desire to change, adapt, or repurpose the virtual layer that augments a preexisting physical environment shared by everyone (i.e. reality-based metaverse). We are starting to see some early proof of the concept of that disruption in standalone apps. For instance, nowadays it is possible to process a video of people to simulate that they were speaking in any desired language hardly distinguishable from the original (Chesney & Citron, 2019). Through XR, LMMs, and user input, it would be possible to transform these interpersonal interactions in real-time. These possibilities expand to other realms too. Image synthesis and style transfer could be applied in real-time to the buildings and urban landscape that surround people on demand: adding more greenery, changing the facades look, hiding buildings, or modifying the views from their window at home or at the office (Mugita et al., 2023). In virtual-based metaverses, the possibilities would be even further due to the lack of physical world constraints. The easier implementation of these powerful generative techniques in purely virtual worlds is already showing their first examples (Hill, 2023). This creates endless changing cities customized for every citizen. Potentially nobody would inhabit the same city if it were even now the case (Fig. 2). Altogether, this collective intelligence may help to create and build cities that reflect and combine more effectively their intrinsic diversity.

### An actionable tool

The challenge of UDTs is helping to provide high-quality built environments, more sustainable and resilient, for people. In general, these tools have to serve a purpose, be trustworthy, and function effectively (Bolton et al., 2018). Beyond issues with major integration challenges (Jeddoub et al., 2023), for having a meaningful digital double closely connected to the physical system (Gerber et al., 2019), the human factor is frequently missing. People need to be considered both from (i) the behavioral (i.e. what it means to consider human preferences, cognition, actions, and socio-cultural aspects) and (ii) the interaction point of view (i.e. what and how can be changed in the digital representation, how preferences and opinions are collected, what are the game rules).

UDTs can be effective tools to test out policies and planning decisions without risk at a minimal cost than in physical reality (Dembski et al., 2020; Mahajan et al., 2022). The development of digital representations of complex urban socio-technical systems enriched with immersive methods of participation, interaction, and feedback for people can provide more effective city planning than existing processes for decision-making (Abdeen & Sepasgozar, 2022). Following these needs, such systems should be able to generate urban planning answers informed directly by a continuous feedback loop created from the interaction of citizens with the urban digital double, the future visions, and also interactions among them. Overall, such frameworks should be able to automate at some degree decision-making, data and preferences collection, feedback gathering and aggregation, processing, visualization, and the generation of alternatives with hybrid intelligence (Dellermann et al., 2019; König et al., 2017), transparently and consistently (Lock et al., 2021). It is, with humans involved and participating in the process, potentially supporting consensus building and democratic processes (Dembski et al., 2020; Helbing et al., 2023).

The motivation for these planning tools is two-fold:

i. Cognitively, by raising situational awareness (Shahat et al., 2021) in the present and future, and bridging the knowledge gap for people when informing their opinion and decision-making regarding complex city-making processes, where participation is increasingly more important;
ii. Operationally, by introducing into UDT models a range of hard-to-measure and uncertain human, social, cultural, and psychological aspects in a machine-readable way.

Partially mirroring BIM collaborative environments (Cardoso Llach & Argota Sánchez-Vaquerizo, 2019), without the hierarchical and finalist constraints, UDTs can help to humanly fine-tune the space of suboptimal solutions according to citizens' (and other stakeholders') preference. A common framework, ideally with publicly defined standards, where different stakeholders can build their own instances in a federated way. Common, interoperable, but at the same time able to host and integrate individual interests, variations, and models. Thereby, this framework of hybrid knowledge could overcome single-dimensional utilitarian optimizations to provide a set of solutions that can satisfy reasonably a diversity of requirements, agendas, and needs. It means pluralistic solutions, and diverse scenarios from collective intelligence supported by aligned machine intelligence (Zaremba et al., 2023) that benefit many different people.

### Code is normative and performative

Building codes and other legal texts regulate spatial occupation, land use, and activities that may happen in space. They have a deep impact on the shape, material, densities, and actions that give place to the built (urban, suburban, and rural) environment (Easterling, 2014).

Institutionalized building codes, as a law, have a deep impact on people's lives and perform of urban spaces (Prytherch, 2012). It determines what and what is not allowed in different parts of the city, which activities and land uses are compatible, and up to which point. It defines densities, built-up surfaces, separation between buildings, occupation of plots, maximum heights, materials, views, infrastructure allocation, public services, distribution of housing units, minimal habitational conditions, shadows, views rights, energetic performance, etc. As such, typological, formal, and functional changes in cities can be analyzed as the final expression of changing building codes and standards over time (Bubola, 2021). In this sense, building codes are spatially and socially performative and normative. It sets and primes human behavior. Overall, it determines the many affordances of spaces. Hence, it can enhance or cancel social and behavioral traits and productive economic activities.

Code as software is equally performative and normative. It regulates what can and cannot be done in physical and digital spaces (Dodge & Kitchin, 2005), and how consistency and trust can be built in such systems. Following the idea of the Internet as a predictor of what is yet to come in more immersive, interactive, and embodied virtual worlds coupled with physical reality, the Web is a socially produced space as it is the urban space (Lefebvre, 1974). However, it comes with the additional constraint that its affordances (Gibson, 1979) are fully limited by the underlying code (Proctor, 2021) even in a more restrictive way:

- Computational legalism: While conceptually similar, software code is more rigid, deterministic, and not subject to interpretation as legal code (Diver, 2021).
- Software-based environment: This type of code is radically defining because it defines all that can be done and built in digital spaces as the own existence of such a digital environment relies completely on code.

This sets a completely different implication of the nature of code as a behavior determiner that is not so acute in physical space. Furthermore, code, even more radically in virtual-based digital spaces, is much harder to hack and circumnavigate, as it accounts not only for what would be conventional norms, rules, and assets in the physical world. It defines also what would be equivalent to the simplest physics and natural laws. In sum, algorithmic governance takes a new and almost totalizing meaning: following the logic of smart contracts, they are executed automatically and deterministically, all the time, permeating every single aspect of these environments, from interactions, pseudo-physical laws, presence, aesthetics, actions. This requires all these elements in tangible, countable, and tokenizable, either for control and ownership purposes, or just to reconstruct digitally some, already lost, sense of aura, value, and authenticity (Kalpokas, 2023; Walter, 1936), if not to retrofit digital abundance with digital commodification (Horkheimer & Adorno, 1947) and a form of simulated scarcity (Bocquillon & Loon, 2022). This power is even more relevant given the known persuasive power of computational frameworks such as the polarizing effects of social media, or by influencing human behavior and opinions such as the case of dark patterns (Fagan, 2024; Reviglio & Agosti, 2020).

Considering (i) the physical, situated anchoring of these UDTs, (ii) their envisioned relevance for governance in these locations, and (iii) the participation of individual and collective (i.e. companies, governmental bodies) stakeholders to exchange their opinions, interests, and concerns, the agency, ownership and sovereignty of these systems cannot be ignored. The convenient outsourcing of the physical infrastructure that supports these cyber-social systems cannot be separated from the governance and policymaking process that they are supposed to support. Also, while digitizing aspects of daily life may save energy, human, and material resources, it comes with the tradeoff of ramping up the resources needed to keep up this physical IT infrastructure. The current development of socio-technical systems for participatory governance and co-creation in cities generates a paradoxical, and potentially risky, situation. To be really actionable and operationalizable these systems need to increase their level of complexity, which simultaneously require relying on larger resources and humongous computing infrastructures that do not belong to the instances that they are supposed to support. They escape public accountability and control. On the contrary, they depend on very few extremely powerful technological players, privately-owned, and market-driven. This compromises sovereignty, self-determination, and the definition of public good (Gstrein, 2023), together with technical risks to centralization and concentration of service providers. What kind of digital illusion is a decentralized and federated metaverse whose needed physical infrastructure depends on a few players?

This ubiquitous and pervasive presence of code and data expands the inherent risks already identified for data-driven smart cities such as dataveillance, geo-veillance, anonymization and re-identification, obfuscation and reduced control, and empty or absent notice (Kitchin, 2016). Also, it includes other risks that are generally associated with data-driven systems such as data and algorithmic biases related to quality, transparency, accountability, representability, agency, and fairness. While decentralized approaches promise to create a self-organized and self-regulated governance scheme, fundamental concerns remain as they may be necessary but not sufficient conditions for ensuring this (Edelman, 2021; Goldberg & Schär, 2023), nor may be participation alone sufficient. Regulatory strategies may be needed (Rosenberg, 2022; Suffia, 2023) and they could help to establish standards and common practices that may help to solve issues related to interoperability and scalability.

### Limits and risks of Urban Digital Twins

UDTs can be powerful tools for planning and simulation. They provide solid support for civic engagement that contributes to more effective decision-making processes. However, their implementation, particularly the deeper that immersive virtual environments are implemented, leads to societal, legal, and ethical risks that can enhance social divisiveness among other issues.

At a very basic level, validation is one of the main concerns. Starting from the acknowledgment of the limitation of the data itself, and their known biases, how can we make sure that the model matches the real dynamics and functioning of the city? This validation process becomes even more complex when considering the most uncertain components, such as human behavior and cognition. Ensuring that these models capture the diversity and unpredictability of social systems is a significant challenge (Caldarelli et al., 2023). However, these systems usually rely on automatic data-driven algorithms and processes without human intervention. The idea that more data will provide more efficient and better decisions necessarily builds on top of massive data collection. Immediately, this raises the question of ownership, agency, and access to data, which ultimately is another enhancer of social divisiveness (Andrejevic, 2014). This expands the whole need for consideration of the human factor beyond purely technical and functional requirements. The benefits of UDTs are not automatic and require a multi-disciplinary approach that takes into account a socio-technical perspective (Kitchin, 2015; Nochta et al., 2021).

The need for data, and its own commodification, force the virtualization of every single asset or aspect into the digital realm, including human behavior, thoughts, and emotions (Bibri & Allam, 2022). Precisely, what is hard, uncertain, or intangible in the physical world needs to be tangible, quantifiable, and computable in the digital world, making every single action easier to monitor in virtual environments, and strengthening trends of surveillance capitalism (Zuboff, 2023). Or what can be more concerning: constraining and nudging human behavior and agency to match the parameters of what can be modeled and measured. An immersive virtual environment may be the perfect realization of a data-controlled and measurable environment: a self-fulfilling prophecy where now everything is, and needs to be for being, measurable, tractable, tokenizable, and monetizable. Virtual environments may provide societal and cognitive advantages (Graham et al., 2022; Hutson, 2022), although they deepen on existing concerns regarding privacy, transparency, accountability, freedom, and fairness in connection with massive surveillance and data –and algorithmic—bias (Helbing & Argota Sánchez-Vaquerizo, 2023). Furthermore, the metaverse in its different implementations can expand the already existing unrealities that people decide to live daily (Eco, 1986). As a consequence, it constitutes a transhumanist escape (Barachini & Stary, 2022) to disregard and ignore the constraints and problems of our physical world (Bojic, 2022; Han et al., 2022; Pal & Arpnikanondt, 2024).

Also, an intense dependence on software makes it vulnerable to the own constraints of how we have created our computer-based technology (Leveson, 2012), including security and obsolescence issues (Strickland & Harris, 2022). VR studies have been proven to be useful for applications in many fields and tasks (Dubey et al., 2018; Sanchez-Sepulveda et al., 2019; Whyte & Nikolić, 2018). However, while advances regarding vision, haptics, and other senses are an active field of research, the whole cognitive process of embodiment and presence may require more sophisticated, and potentially more neurologically invasive approaches (Pisarchik et al., 2019), to be able to match the experience of physical presence. Natural and social conventions that exist in the physical world, and the tangibility of people and goods exchange and ownership need to be redefined by code from scratch in virtual environments (Huynh-The et al., 2023; Zwitter et al., 2020). If blockchain would make trust unnecessary for human relations and interactions (Edelman, 2021), it is the metaverse where it can really flourish, due to the lack of real embodiment. Hacking the physical world is harder and more obvious; humans are more adapted to detect it. Since it is code-based, it presents easier opportunities for inconspicuous hacking, affecting the people who inhabit it as avatars. For instance, it is relatively easy to notice somebody hiding in our vicinity or some device trying to spy on us, but what happens when the endless data streams needed for defining every single interaction in the digital world can be incepted or other identities can be completely camouflaged into the environment? What kind of countermeasures should be implemented? (Lee et al., 2021; Wang et al., 2023).

Digital doubles, including metaverses, are ultra-plastic and endlessly replicable: everything can be changed, cloned, and transformed, at a minimal cost. This provides advantages for adaptability, diversity, and testing in comparison with the tectonic, physical, heavy, and tangible physical environment. However, this flexibility makes metaverses require clear rules and procedures for interaction that need to be designed to be useful and remain coherent and consistent (Hudson-Smith & Batty, 2023). Each representation of a city within a UDT, with its myriad of alternative scenarios and changes and modifications due to personal preferences, is different, which challenges cohesion and integration. They may be mirroring only partial aspects of the physical environment (Helbing, 2013). They may respond to non-existing alternative scenarios to be explored and analyzed. They may be customized variations chosen by individuals to match their needs. If this multiplicity would not be enough, on a more fundamental level, each of us does not experience the same environment in the same way. Each person and social group focus, prioritizes, and is affected differently by different components, layouts and features of the city. Even if the experience of each social group or individual is always partial in the city, the whole set of urban dwellers identifies the whole as a single entity. This effortless replicability can boost the creation of individual versions of an XR leading to an intense digitization of urban life makes easier to extend spatial segregation and accessibility restrictions to citizens (Cardullo et al., 2019). Making significant parts of urban life only accessible through technology may widen existing digital exclusion issues. This would hinder the attempt to create a common united identity within a city expanded in the digital world. We will face integration, interoperability, and scalability issues (Cheng et al., 2022) to ensure togetherness.

Fostering citizen participation is not an automatic fix for the shortcomings of computational methods, nor for *unblackboxing* some of these technologies (Sloane et al., 2022). Participation by itself won’t solve immediately political challenges related to the prioritization of goals or defining what is the common good (Zografos et al., 2020), nor the use of digital twins will take over the construction of democratic consensus despite its power to smooth communication between stakeholders (Yamu et al., 2023). Thereby, new and richer methods for assessing the quality of this digital participation need to be explored and developed (Ataman et al., 2022). Citizen participation is a key element for sustainable urban development (Bouzguenda et al., 2019) and UDTs facilitate the decision-making process by easing the simulation of future scenarios and policies (Mahajan et al., 2022), by making their results more understandable, and by smoothing and enriching exchange between stakeholders (Haraguchi et al., 2024). Even further, these complex cyber-physical systems can perpetuate knowledge, agency, and power inequalities. Thereby, it is needed a socio-technical and participatory perspective in the development of these tools, beyond pure technical feasibility, enhancing aspects of trustworthiness and purpose, which often require very local and situated knowledge, encompassing also organizational culture to reach actionable levels of trustworthiness and legitimacy (Bolton et al., 2018; Nochta et al., 2021). This can ease the challenge of integration and acceptance of shifts in policymaking. Many of the existing initiatives focusing on marketing, business, and finance may be rendered deceptive due to a poor functional experience and lack of valuable purpose within these domains beyond the initial hype. However, this highlights the opportunity and promising value of urban metaverses based on UDTs conceived as co-creation, exchange, and sharing environments oriented to governance, policy, and planning.

By now, in the end, the digitalization of the urban realm as we have known until now has ended up being very different. Instead of developing bottom-up participatory approaches to empower people via open-source technology, it was taken over by corporate, capitalist, and market-driven approaches (Greenfield and Kim 2013). This perpetuates power inequalities (Egliston & Carter, 2021) and control with social consent (Han, 2017). Finally, UDTs, understood as a technocratic continuation of smart cities, are not quite there yet regarding community participation (Axelsson & Granath, 2018), but neither regarding standardization, interoperability, and scalability (Cheng et al., 2022; Shahat et al., 2021). From a societal and cultural point of view, the outmost question remains about how important, needed or even required would be these technologies to live in cities. Beyond being a nice and convenient add-on to daily life, or being a potential enhancement of social and experiential segregation, it is yet to be proven if their functionality, purpose, and trustworthiness can make them fundamental components of daily life, marginalizing people who are not participating of them (Cardullo et al., 2019).

## Concluding remarks

This article tries to reframe the concept of UDTs beyond a purely technical and engineering perspective. It presents a broad overview of existing examples of UDTs. Some of them, which are not usually considered as such, may be even more accomplished implementations of this concept. As a result, it is possible to map key features of ideal UDTs, which are currently present only partially in different cases. From these fragmentary components it is possible to build an image of future UDTs. This allows for anticipating positive and negative outcomes, particularly in connection with citizen participation and human interaction because of the possibility of offering multiple interactive and usable urban digital experiences. Although many potential beneficial effects for better more inclusive and sustainable cities can be expected, it highlights the many risks not only associated to the usual concerns on privacy, freedom, and fairness common when dealing with big data, but also stress the risk for increasing societal divide. Hence, it expands the concept to human-centric considerations to explore what is missing to generate benefits for a bigger number of individuals.

Our current UDTs do not fulfill our idealized concept of mirrored representations of our physical world systems with automated exchange of information. As such, that accomplishment would mean that both entities would be merged into a single system. On one hand, on a conceptual level, it may be impossible to have such a system. On the other hand, from a practical perspective, it would be our loss, as one may want to explore alternative scenarios and possibilities, which are out of our existing world. It means, decoupling the twin for learning something new. From a technical point of view, having transferred the idea of digital twins from the mechanistic manufacturing and spatial engineering worlds to complex urban systems missed the point of externalities, and above all, the human factor in it, which involves cultural, social and psychological aspects hard to account and encode into a digital code-based system. What may be valuable is precisely not trying to fit a deterministic forecast to complex systems that involve socio-technical factors. In this sense, engaging citizens as humans-in-the-loop may be more actionable, powerful, and effective to benefit as many people as possible. Digital twins can be more powerful for their exploratory power for shaping thinking and helping us to pose questions (Kac, 1969), than for their predictive capabilities for scenarios and operations.

Despite its immense value for inclusive and participatory governance in cities, and for fostering quality of life and sustainability of cities, we need to be aware of the potential risks of the further development of such systems in immersive XR environments. The experience of being immersed, interacting, and sharing leads to urban metaverses, which as any other technology, may have dual uses. On one hand, it may expand the possibilities to explore alternative scenarios that may be experienced before being implemented and support collective intelligence for more effective policymaking and city planning. Alternatively, it can be used for tokenizing and segregating (even further) cities and societies. Our physical, built environment will expand through XR. This is an opportunity to reflect and respond more effectively to human diversity. At the same time, chances of fostering social divisiveness will increase with minimal effort.

Contrasting with an abundance landscape provided by automated multi-purposed AI, human experience will be comparatively more scarce and more valuable over time. The scalability and reproducibility of software are bidirectional: many different copies, versions, and alternatives of virtual environments can be created, in the same way that many different versions of augmentations can be done to populate the same physical environment. However, it is unclear how many of us will be sharing these spaces.

## Figures and Tables

**Table 3:**
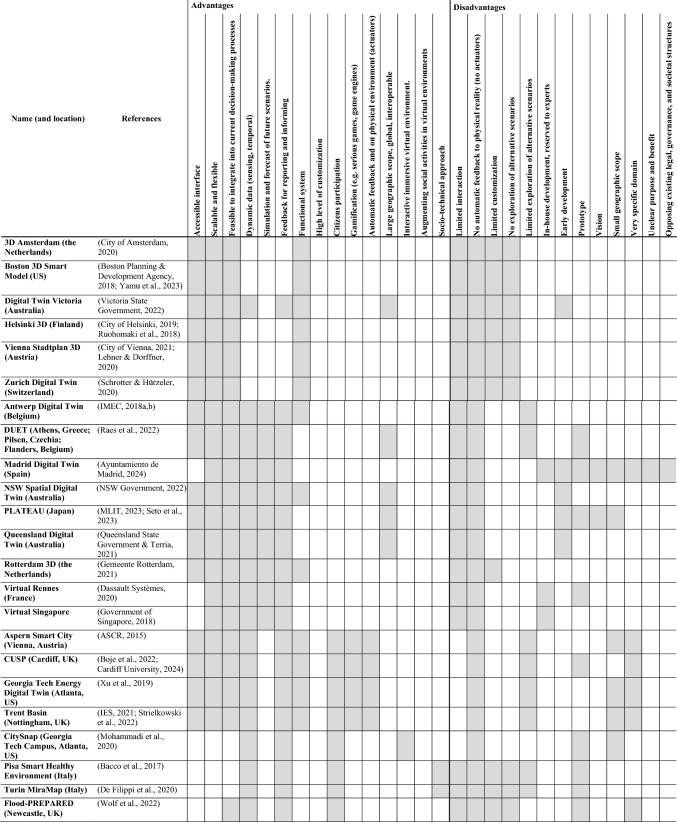

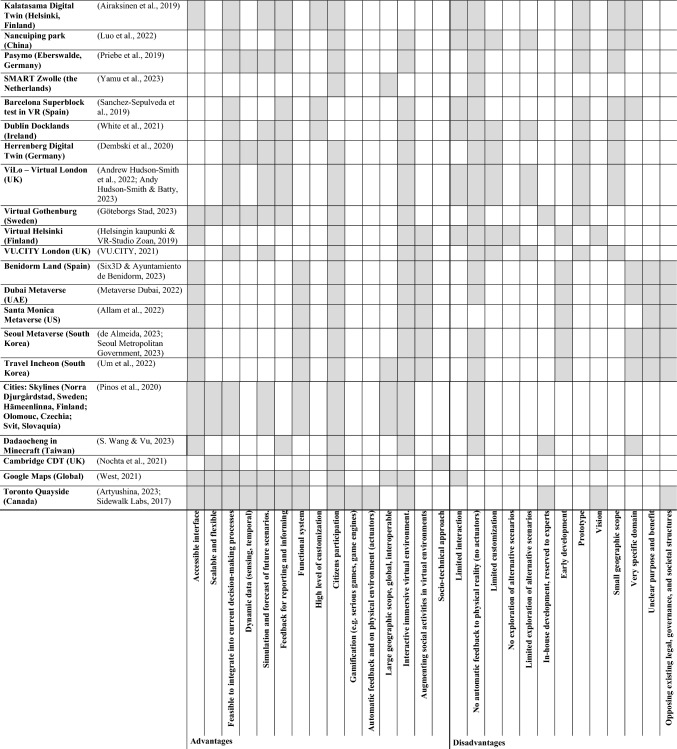
Comparative analysis with shared advantages and disadvantages of the considered examples of Urban Digital Twins

Examples are sorted based on their similarities following the analysis in Section Examples of Urban Digital Twins

## Appendix

### Characterization of the analyzed examples of Urban Digital Twins

See Tables 2 and 3

**Table 2:** General description of the analyzed examples of interactive, functional and situated Urban Digital Twins

Name (and location)	Scope							Stage	Access	References	Description
	Viz	GIS	Environment	Participation	Planning	Services	Immersive				
3D Amsterdam (the Netherlands)	X				X			Deployed WIP (Work-in-Progress)	Online	(City of Amsterdam, 2020)	City government interactive 3D viewer for existing geospatial datasets, detailed buildings, urban plans, and perform some basic environmental simulation such as sun lighting
Antwerp Digital Twin (Belgium)	X		X					Deployed WiP	Online demo	(IMEC, 2018a, b)	Prototype of the city government interactive 3D viewer with dynamic data integration that allows for simulating the impact on traffic, air quality, or flooding due to disruptive events
Aspern Smart City (Vienna, Austria)		X	X	X	X	X		Deployed	Restricted	(ASCR, 2015)	Management tool for real-time urban data and smart energy utilization in a newly built district
Barcelona Superblock test in VR (Spain)				X	X		X	Prototype	Restricted	(Sanchez-Sepulveda et al., 2019)	Immersive VR prototype to test how active citizen participation can be promoted in the context of collaborative urban design
Benidorm Land (Spain)				X		X	X	Deployed	Online	(Six3D & Ayuntamiento de Benidorm, 2023)	Fully VR environment, geometrically accurate based on the physical city, to explore, and promote tourism and online events
Boston 3D Smart Model (US)	X	X			X			Deployed WiP	Online	(Boston Planning & Development Agency, 2018; Yamu et al., 2023)	City government interactive 3D viewer for existing geospatial datasets, to support and keep track of planning and urban design projects and enhance exchange and participation
Cambridge CDT (UK)		X		X	X			Developed	Restricted	(Nochta et al., 2021)	Introducing socio-technical approaches in the development of a specific urban digital twin to reach satisfactory levels of functionality, trust, and purpose for a local context
Cities: Skylines (Norra Djurgårdstad, Sweden; Hämeenlinna, Finland; Olomouc, Czechia; Svit, Slovaquia)	X		X	X	X			Deployed WiP	Restricted	(Pinos et al., 2020)	Workshops for citizen participation in urban planning through gamification using existing game engines
CitySnap (Georgia Tech Campus, Atlanta, US)	X			X	X	X		Prototype	Restricted	(Mohammadi et al., 2020)	XR application for providing citizens´ feedback on the functioning of urban aspects, sharing, and visualizing the city´s state based on crowdsourcing and sensing data, with a case study for a university campus
CUSP (Cardiff, UK)	X	X	X			X	X	Developed	Online demo	(Boje et al., 2022; Cardiff University, 2024)	Decision support platform for smart energy use, monitoring, and optimization based on immersive 3D visualization and semantic data
Dadaocheng in Minecraft (Taiwan)	X	X	X	X	X			Developed	Restricted	(Wang & Vu, 2023)	Virtual environment for participation in planning and urban reactivation through gamification using existing game engines
Digital Twin Victoria (Australia)	X	X			X			Deployed	Online	(Victoria State Government, 2022)	Regional interactive 4D geospatial data viewer, with data in near real-time to support improved decision-making
Dubai Metaverse (UAE)				X		X	X	Deployed	Online	(Metaverse Dubai, 2022)	Fully VR environment, geometrically accurate virtual double of the city to purchase virtual land and enhance tokenized virtual economy without a direct linkage to the real physical world more than for linking tokens, marketing, and business promotion-related actions
Dublin Docklands (Ireland)	X		X	X	X			Developed	Restricted	(White et al., 2021)	Virtual environment mirroring a part of the city to run behavioral user-based experiments regarding different interventions in the urban space: construction of new buildings, new green areas, reporting issues, flooding, and crowd acceptance
DUET (Athens, Greece; Pilsen, Czechia; Flanders, Belgium)			X	X	X		X	WiP	Restricted	(Raes et al., 2022)	Pilots of a scalable architecture framework able to contain models and data synchronized with the mirrored physical environment and integrate through APIs different models to perform what-if analysis related to traffic, air quality, or noise pollution
Flood-PREPARED (Newcastle, UK)		X	X					Developed		(Wolf et al., 2022)	A real-time city-wide sensing system for precipitation monitoring to simulate flood hazards and support multi-agency emergency management and coordination
Georgia Tech Energy Digital Twin (Atlanta, US)	X			X	X			Developed	Restricted	(Xu et al., 2019)	VR immersive integrated eco-feedback system to interact with real-time energy consumption on a university campus
Google Maps (Global)	X			X				Deployed WiP	Online	(West, 2021)	Global privately owned and accessible mapping and navigation interactive platform with real-time geospatial data integration that supports a wide variety of services for data visualization, analysis, and simulation
Helsinki 3D (Finland)	X		X	X				Developed	Online	(City of Helsinki, 2019; Ruohomaki et al., 2018)	City government interactive 3D viewer for existing geospatial datasets, detailed buildings, urban plans, and perform some basic environmental simulation such as climate and energy performance
Herrenberg Digital Twin (Germany)	X		X	X	X			Developed	Restricted	(Dembski et al., 2020)	Prototype for participatory urban planning in a small city that included a VR immersive 3D environment, diverse geospatial data, and mobility and wind flow simulations
Kalatasama Digital Twin (Helsinki, Finland)	X	X	X	X	X			Developed	Restricted	(Airaksinen et al., 2019)	An open 3D geometrically realistic model for a district of Helsinki as a platform for application development, for interacting with the residents, to analyze and simulate plans, and for new services, processes, and practices
Madrid Digital Twin (Spain)	X	X	X		X	X		Deployed WiP	Restricted	(Ayuntamiento de Madrid, 2024)	City government platform that integrated multi-source and multi-format data, IoT, and AI for enhanced urban planning, public services, and policymaking, fostering stakeholder collaboration and scalable integration of urban smart applications
Nancuiping park (China)	X	X		X	X			Developed	Restricted	(Luo et al., 2022)	Comparison between a digital twin and a physical model as a resource for participatory design workshop for a park
NSW Spatial Digital Twin (Australia)	X	X			X			Deployed	Online	(NSW Government, 2022)	Regional interactive 4D geospatial data viewer, with near real-time data and BIM/CIM integration capabilities, to support improved decision-making
Pasymo (Eberswalde, Germany)	X			X	X		X	Developed WiP	Restricted	(Priebe et al., 2019)	Multiplatform (on desktop and VR) academic research project to enhance participatory urban planning capabilities with the use of advanced data visualization and simulation of alternatives
Pisa Smart Healthy Environment (Italy)			X	X				Prototype	Restricted	(Bacco et al., 2017)	IoT ecosystem with fixed and mobile sensors, collecting and processing geo-referenced environmental data, enhanced by citizen participatory sensing and data for improved urban air quality and micro-climatic monitoring
PLATEAU (Japan)	X	X	X	X	X	X		Deployed WiP	Online	(MLIT, 2023; Seto et al., 2023)	A nationwide platform for 3D urban models integrating data on mobility, demographics, economy, and business ready to perform advanced simulation and analyses for planning, disaster prevention, and public services
Queensland Digital Twin (Australia)	X	X			X			Deployed WiP	Online	(Queensland State Government & Terria, 2021)	Regional interactive 4D geospatial data viewer, with near real-time data and BIM/CIM integration capabilities, to support improved decision-making
Rotterdam 3D (the Netherlands)	X	X		X				Deployed WiP	Online	(Rotterdam, 2021)	City government interactive 3D viewer for existing geospatial datasets, detailed buildings, urban plans, and perform some basic environmental simulation such as sun lighting
Santa Monica Metaverse (US)						X	X	Developed	Online app	(Allam et al., 2022)	AR application, gamifying, and incentivizing urban exploration and interaction with the environment to promote digital and local economy and trade
Trent Basin (Nottingham, UK)	X	X	X	X		X		Deployed	Restricted	(IES, 2021; Strielkowski et al., 2022)	Management tool for real-time urban data and smart energy utilization in a new urban development
Seoul Metaverse (South Korea)				X	X	X	X	Deployed WiP	Online	(de Almeida, 2023; Seoul Metropolitan Government, 2023)	Government-led initiative to recreate the physical space in an immersive VR environment to expand possibilities of leisure, cultural events, public services, and economy activity
SMART Zwolle (the Netherlands)	X	X	X	X	X			Developed WiP		(Yamu et al., 2023)	City government interactive platform to report and aggregate existing geospatial datasets, detailed buildings, urban plans, sensing, participatory initiatives, and monitor different aspects of urban life
Toronto Quayside (Canada)	X			X	X	X		Discontinued	Restricted	(Artyushina, 2023; Sidewalk Labs, 2017)	Full algorithmic governance and policymaking scheme based on automatic data-driven processes
Travel Incheon (South Korea)						X		Deployed WiP	Online app	(Um et al., 2022)	Smart tourism metaverse with 'AR Incheon' for augmented reality experiences and 'Incheoncraft' for immersive virtual exploration using Minecraft, blending history with interactivity
Turin MiraMap (Italy)		X		X	X			Developed	Restricted	(De Filippi et al., 2020)	City platform for citizens' engagement in policymaking and planning; to report issues, express opinions, collect proposals, and map and visualize all these contents
Vienna Stadtplan 3D (Austria)	X	X		X				Developed	Online	(City of Vienna, 2021; Lehner & Dorffner, 2020)	City government interactive 3D viewer for existing geospatial datasets, detailed buildings, urban plans, and perform some basic environmental simulation such as sun lighting
ViLo – Virtual London (UK)	X	X		X			X	WiP	Restricted	(Hudson-Smith & Batty, 2023; Hudson-Smith et al., 2022)	Immersive platform to visualize and aggregate diverse geospatial data to foster exchange, analysis, participation, and exploration of urban plans
Virtual Helsinki (Finland)				X			X	Developed	Online	(Helsingin kaupunki & VR-Studio Zoan, 2019)	VR environment, geometrically accurate and realistic based on the physical city, to explore, and promote tourism and online events. Also available as a Minecraft version as a gaming environment
Virtual Gothenburg (Sweden)	X	X		X	X		X	WiP	Restricted	(Stad, 2023)	City government interactive platform to visualize and aggregate existing geospatial datasets, detailed buildings, and urban plans, collect proposals, and engage citizens in urban issues, planning, and policymaking tasks, while supporting tests and simulations for these plans
Virtual Rennes (France)	X	X	X		X			Developed	Restricted	(Dassault Systèmes, 2020)	City government interactive 3D viewer for existing geospatial datasets, detailed buildings, urban plans, and perform some basic environmental simulation such as sun lighting
Virtual Singapore	X	X	X		X			Deployed WiP	Restricted	(Government of Singapore, 2018)	City government interactive 3D viewer for existing geospatial datasets, detailed buildings, and urban plans, and perform some disciplinary and domain-specific simulations such as sun lighting, mobility, emergency management, or energy
VU.CITY London (UK)	X	X	X		X		X	Deployed WiP	Restricted	(VU.CITY, 2021)	Viewer for existing geospatial datasets, historical data, urban analytics for planning, integration of new projects and workflows for stakeholders
Zurich Digital Twin (Switzerland)	X	X			X			Deployed WiP	Online	(Schrotter & Hürzeler, 2020)	City government interactive 3D viewer for existing geospatial datasets, detailed buildings, urban plans, and perform some basic environmental simulation such as sun lighting

Cases are sorted alphabetically
